# A Combinatorial Solution to Point Symbol Recognition

**DOI:** 10.3390/s18103403

**Published:** 2018-10-11

**Authors:** Yining Quan, Yuanyuan Shi, Qiguang Miao, Yutao Qi

**Affiliations:** 1 The School of Computer and Technology, Xidian University, Xi’an 710071, China; ynquan@xidian.edu.cn (Y.Q.); syy960315@163.com (Y.S.); qgmiao@xidian.edu.cn (Q.M.); 2 Xi’an Key Laboratory of Big Data and Intelligent Vision, Xi’an 710071, China

**Keywords:** point symbols recognition, feature extraction, deep transfer training, preprocessing

## Abstract

Recent work has shown that recognizing point symbols is an essential task in the field of map digitization. For the identification of symbols, it is generally necessary to compare the symbols with a specific criterion and find the most similar one with each known symbol one by one. Most of the works can only identify a single symbol, a small number of works are to deal with multiple symbols simultaneously with a low recognition accuracy. Given the two deficiencies, this paper proposes a deep transfer learning architecture, where the task is to learn a symbol classifier with AlexNet. For the insufficient dataset, we develop a method for transfer learning that uses a MNIST dataset to pretrain the model, which makes up for the problem of small training dataset and enhances the generalization of the model. Before the recognition process, preprocessing the point symbols in the map to coarse screening out the areas suspected of point symbols. We show a significant improvement over using point symbol images to keep a high performance in being able to deal with many more categories of symbols simultaneously.

## 1. Introduction

In resource development, engineering construction, and military defense [1], nowadays, many scientific research institutions and researchers have proposed some algorithms for the recognition of point symbols in color topographic maps.

Recognizing a map consisting of points, lines, and surface symbols is equivalent to identifying various points, lines, and surface symbols. Furthermore, since the line symbols are composed of basic point symbols through multiple combinations, automatic recognition of the point symbols is the core for the entire map symbol recognition. At present, the point symbol is collected by a manual method in a topographic map, that is, the position determines by manual observation and the corresponding attribute information is input. A large number of practical work shows that the manual collection method has many obvious shortcomings, such as low collection efficiency and accuracy, and a large workload of the collector. At the same time, due to the lack of effective detection means, the extraction and missed mining of point-like symbols in color topographic maps also occur from time to time, which seriously affects the efficiency and reliability of digitization of color topographic maps. Therefore, for a large number of color topographic maps, it is unrealistic that the point symbol is wholly accomplished using manual acquisition on the topographic map. Consequently, it is especially important to study how to accurately identify the point symbols in the map for improving the efficiency of map digitization.

These existing research works can be divided into the following five categories: (1) Template matching [2], the symbols are identified by comparing the similarities between the candidate symbols and the template, comparing the similarity criteria that depend on the matching. This method must take into account the deformation of the corresponding template. Based on the method, Qi focuses on clustering-based band selection and proposes a new framework to solve band selection [3]. However, template matching cannot adapt to the various changes such as scaling and local deformation. This method is more suitable for linear feature separation [4]. (2) Statistical [5], the core of which is the classifier and the extraction of the distinctive features. This method is applied to many aspects, such as the identification of labels on maps [6], face recognition under lighting conditions [7], etc. (3) The structure method [8], which is based on the structural characteristics of the recognition object to simplify an intricate pattern into several sub-patterns and recognizes the complex pattern via recognizing each sub-pattern. A fusion of statistical methods and structural methods [9] has been proposed, which obtained a good result by tracking and identifying the profile of the object based on hidden Markov model(HMM). An interesting method [10] has been proposed by decomposing each pixel and the spatial neighborhood into a low-rank form, and the spatial information can be efficiently integrated into the spectral signatures. This way relies relatively on symbol extraction, for the complex topographic features, the extraction results are inevitably less than satisfactory. (4) Mathematical morphological that is not only used in the processing of topographic map information, including symbol recognition [11] and boundary separation [12] but in license plate recognition [13]. However, the mathematical morphological is inefficient, computationally intensive, and takes a long time to process. (5) Neural network [14], which is regarded as the complex network that forms by interactions of many neurons. The learning ability of the neural networks is similar to the human brain, and high adaptability of it can identify the input effectively even if the images have little noise. In addition, the improved back-propagation(BP) neural network is introduced to identify point symbols by some researchers at present [15]. For hyperspectral images, in order to better handle the high-dimension problem and explore abundant information, a general end-to-end 2D convolutional neural networks(CNN) framework is presented [16]. However, there are still some drawbacks, causing problematic training and a low recognition rate for neural networks, such as the problem of gradient dispersion and the shallowness of network training. For these reasons, the application of neural networks is limited.

Therefore, our research focuses on high recognition accuracy while dealing with multiple symbols simultaneously. Recently, the emergence of deep learning achieves identifying numerous symbols simultaneously to some extent. One of the factors for the triumph of Deep Learning is the massive data. However, the available map data for point symbols recognition is limited by the privacy of the information it represents, resulting in less data for training and learning, which leads to lower identification. Thus, this paper proposes a deep transfer learning architecture based on AlexNet.

In this paper, the deep transfer learning architecture based on AlexNet network is pretrained by the MNIST dataset to obtain a preliminary model in the proposed algorithm firstly. In addition, the preliminary model is then trained with the point symbol dataset in depth to get to the final-trained model for the point symbol recognition problem. Furthermore, the region of the suspected point symbol is extracted based on the regional characteristics of the point symbol to accomplish the preliminary prescreening before recognition. Then, the extracted regions of point symbols are identified via the final-trained model to classify point symbols. The recognition method with the deep transfer learning not only improves the accuracy of recognition but also handles multiple point symbols simultaneously. We, therefore, introduce some contributions that are critical to the success of our methods. These include:

(1) We experimentally show that the method of deep neural networks is good feature learning and classifying machines that model recognizes multi-symbols simultaneously.

(2) We develop a way of transfer learning that uses an MNIST dataset to pretrain the model, which makes up for the problem of a small training dataset and enhances the generalization of the model.

(3) Taken together, we are able to present a general and robust method. In an extensive set of experiments, we present clear advantages over both the classical and recent method. The remainder of this paper is organized as follows. In Section 2, the challenge of point symbols recognition in a topographic map is introduced briefly. The idea of the algorithm is described in Section 3. Comparison experiment results are presented in Section 4. Finally, conclusions are given in Section 5.

## 2. Related Works

### 2.1. Point Symbols in the Topographic Map

Point symbols represent the element needed to be displayed in the topographic map such as stadiums, power stations, and oil depots, but are unable to present its target geometry in the scope of the topographic map. In the topographic map, each element is distinguished by a different color, and the point symbol is black generally.

**Figure 1:**
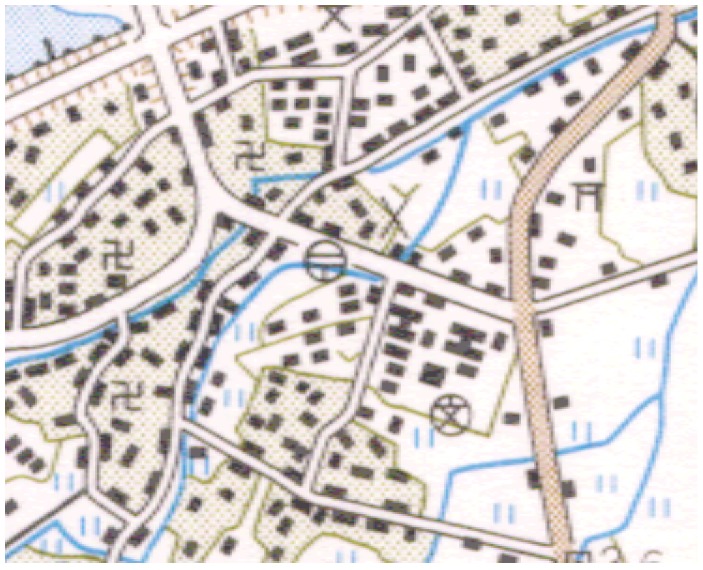
The point symbols in the topographic maps.

Researchers proposed lots of methods to recognize the point symbols and devoted a lot of effort to improving the recognition accuracy. In [17], by analyzing the topological properties of the symbolic graph, Euclidean numbers and moment features are first calculated. Then, point symbols are classified according to the processing of multi-classifiers and the prior knowledge. In [18], point symbols are identified according to the various characteristics extraction of point symbols including the moment invariant based on the invariant moment theory. In most research, the recognition of the point symbols is disturbed by the similarity in size and the complexity of the backgrounds in the topographic map. At present, most of the algorithms are only processed for a single symbol once. However, in some topographic maps with low-quality, such as Figure 1, the symbols cannot be extracted accurately, and it remains a great challenge for the symbol recognition in the complicated situation. Additionally, the similarity among the point symbols in size and the regularity of the shape are disruptive for extracting and identifying a single symbol in most of the current algorithms.

Furthermore, the point symbols are shown in Figure 1, the characteristics of which in the topographic map are summarized as the following [19,20]:

(1) The shape of the symbol is relatively fixed and does not change with their location.

(2) Point symbol has a specific direction which is perpendicular to the southern edge, except for several rotatable symbols.

(3) The shape of the symbol was regular and most symbols are made of a simple geometric pattern.

(4) Point symbol is small and the sizes of the symbol are similar to each other.

Based on the above statements, we embrace these observations effectively and aim to identify more point symbols once. Compared with most works, the biggest highlight of ours is to judge whether it is a point symbol based on the similarity between the size of symbols and the regularity of the shape. Furthermore, most algorithms on point symbol recognition can only handle a single symbol at once, which results in inefficient identification. Thus, the deep transfer learning is introduced to identify the point symbol in this paper, where the goal is to handle more than one symbol with a higher accuracy simultaneously. The effect of the rotation of the point symbol extraction is small because the direction of the symbol is substantially fixed so that the effect of rotation can be ignored.

### 2.2. Application of Deep Learning in Object Recognition

In recent years, the rapid development of artificial intelligence has set off a new wave of Deep Learning. Geoffrey and his students refresh the records on Image Net classification, on which Deep Learning laid a crucial status in the field of image recognition successfully. In 2012, based on deep learning [21],the system of Google Brain led by Stanford professor Andrew Ng and Jeff Dean was able to learn and identify cats automatically. This project has aroused a great sensation in academia and industry stimulating the upsurge of Deep Learning in the world.

The real strength of Deep Learning is that its characteristics learning is driven by data, and learns the characteristics from the data directly without factitious design. Meanwhile, it is also impeded by the massive data. Deep Learning is widely applied to various fields of computer vision [22], vehicle detection [23], and gesture recognition [24] and so on. Excitingly, it is the first time that Deep Learning is introduced to the recognition of the point symbols in topographic maps. Due to strong classification, the associative ability of neural networks and splendid adaptation, self-organization, self-learning and fault tolerance, a high performance of the symbols recognition is shown for the problem of mutual adhesion, mutual glands, and incomplete and deformations in the map. In contrast, common methods for these issues have difficulty achieving satisfactory recognition accuracy. However, the deep neural network has a deep hierarchy for obtaining the hierarchical extraction of the features to avoid the most difficult problem, which is feature extraction for the symbolic recognition. Meanwhile, the recognition speed is fast due to the parallel processing of neural networks and the adaptive adjustment of network weights.

Therefore, the deep neural network is introduced in the proposed algorithm. The training data is one of the essential limits for the deep learning. The inefficient data will impede the training to desolate the accuracy. For the point symbols, owing to the map information possibly containing the national secret, and the amount of available map data being small, the amount of training data for the neural network is significantly limited, leading to a lower accuracy of recognition. Based on the transfer learning, the preliminary AlexNet model trained by the MNIST dataset, the final-trained classifier model that is used to recognize the symbols is obtained by inputting the point symbols into the preliminary AlexNet model. Importantly, the final-trained model has learned the characteristics of the point symbols further. The method is more applicable for learning the characteristics of the point symbols. Finally, the final-trained model is regarded as the classifier used to recognize the point symbols.

## 3. Method

As introduced earlier, the CNN networks are one of the popular ways to solve recognition. We plot the algorithm framework of our two-stage architecture for point symbol recognition, as shown in the table. It consists of three stages: the prescreening stage, the learning stage, and recognition. We firstly elaborate on the design of the prescreening stage and the learning stage, and then introduce implementation details of the whole networks.

**Algorithm Framework**       Procedure of Point Symbol Recognition
**Step 1 Prescreening the topographic maps.**
      Step 1.1 The topographic map is processed to extract the black sub-layouts.      Step 1.2 Based on the method of judging the minimum bounding rectangle, the connected region of suspected point symbols should be obtained.
**Step 2 Training the model.**
      Step 2.1 Pretraining the AlexNet networks based on MNIST database by transfer learning to obtain the pretraining model.      Step 2.2 Pretaining the pretraining model based on the point-symbols data to obtain the final model.
**Step 3 Recognition of the point-symbols.**
      The test images of point symbols input the final model to test the recognition accuracy.

In this paper, a distinctive recognition algorithm of point symbols is designed based on the AlexNet. We propose a new method to recognize more than one point symbol at once automatically, which learns the characteristics of point symbols via the training of Deep Learning directly after the preprocessing. The main idea of the proposed algorithm is to prescreen based on the regional characteristics of the point symbols dataset and pretrain the AlexNet based on a MNIST dataset at first. Next, AlexNet is trained again based on the point symbols to learn the characteristics of point symbols further so that multiple point symbols can be handled with a high accuracy simultaneously.

### 3.1. The Prescreen Based on the Characteristics of Point Symbols

Compared with other annotation symbols, the point symbols are similar to each other in size and the length-to-width ratio of the circumscribed rectangle, respectively. According to the characteristics mentioned above, the suspected connected region can be extracted. Therefore, the main task for the preprocessing is the first to screen the connected region of the suspected point symbols.

**Figure 2:**
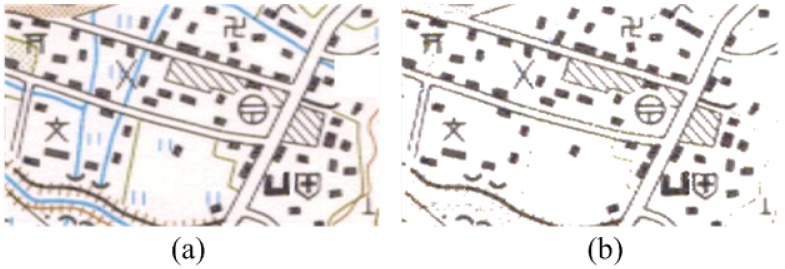
The figure shows the comparison of map changes after color segmentation, as follows: (**a**) the original image of the topography map; (**b**) the sub-layout images of the topography map.

**Figure 3:**
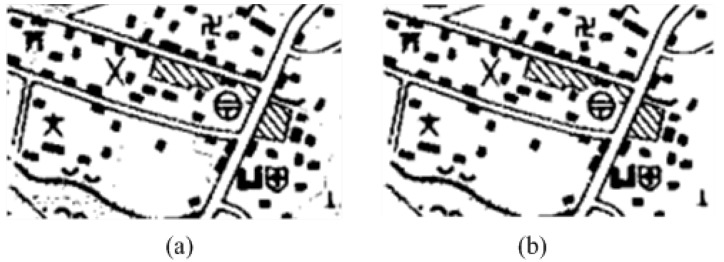
This figure plays the comparison of map changes after prescreening. They are listed as (**a**) description of figure after binarization; (**b**) description of the figure that extracts the black sub-layouts and eliminates the noise.

As shown in Figure 2, the original image of the color topographic map is displayed. Because there are so many elements that interfere with the inability to extract connected areas of suspicious point symbols, before prescreening the point symbols’ connected region, firstly the topographic map is reprocessed to reduce the interference such as color and noise, which includes performing color segmentation to extract the black sub-layouts containing the point symbols, the binarization and noise- elimination. In addition, it can be seen from Figure 3.

**Figure 4:**
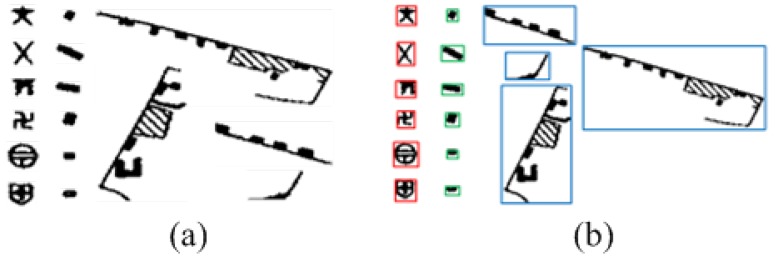
(**a**) the suspected symbols; (**b**) the connected region (CR).

To analyze the characteristics of all point symbols and non-point symbol regions in Figure 3b, both of which are extracted and shown in Figure 4a, in this paper, the regional characteristics as one condition screen the circumscribed rectangle of the suspected point symbols, owing to obvious characteristic differences of the circumscribed rectangle between the point symbols and the non-point symbol in most cases. As shown in Figure 4b, on the one hand, the difference in size among point symbols is small. On the other hand, the circumscribed rectangle of the point symbols in a color topographic map closely resembles the square. The red is the connected regions of point symbols, the blue and the green are the connected regions of non-point symbols in Figure 4b. There are some apparent differences between the red and the blue, but the blue and the green are similar. Thus, the point symbols and the non-point symbols can be distinguished by the difference in size and the shape. When there is an enormous difference between the metric of circumscribed rectangle and the threshold, the symbol is regarded as a non-point symbol such as the blue and the green in Figure 4b. When the metric of the circumscribed rectangle is similar to the threshold, the symbol is regarded as a point symbol temporarily such as the red in Figure 4b. However, there are still some non-point symbols being saved because some similarities exist between the circumscribed rectangle of point symbols and non-point symbol. Thus, subsequent deep transfer learning based on neural network is necessary.

Whether the connected region should be deleted depends on the minimum bounding rectangle of itself. The following steps obtain the minimum bounding rectangle. Firstly, all of the connected regions in the color map are filtered by scanning the black sub-layout. Then, the length-to-width ratio of the connected region is analyzed to choose the minimum bounding rectangle (BR). When the smallest bounding rectangle of CR meets either of conditions shown in Equation (1), the connected region will be judged as a non-point symbol region and the annotation symbol in this region is not regarded as a point symbol. In addition, the corresponding connected region is not considered directly. Otherwise, this connected region is tentatively recognized as a point symbol. The smallest bounding rectangle of the connected region (CC.BR co-infection as follows) gives a criterion for judging shown in Equation (1):

(1) $$\left\{\begin{array}{c} CR.BR.Height>2\times Size∥CR.BR.Width>2\times Size,\\ CR.BR.Height<\frac{Size}{2}∥CR.BR.Width<\frac{Size}{2}. \end{array}\right.$$

CR.BR.Height and CR.BR.Width mean the height and the width of the smallest circumscribed rectangle of the Connected Region, respectively.

**Figure 5:**
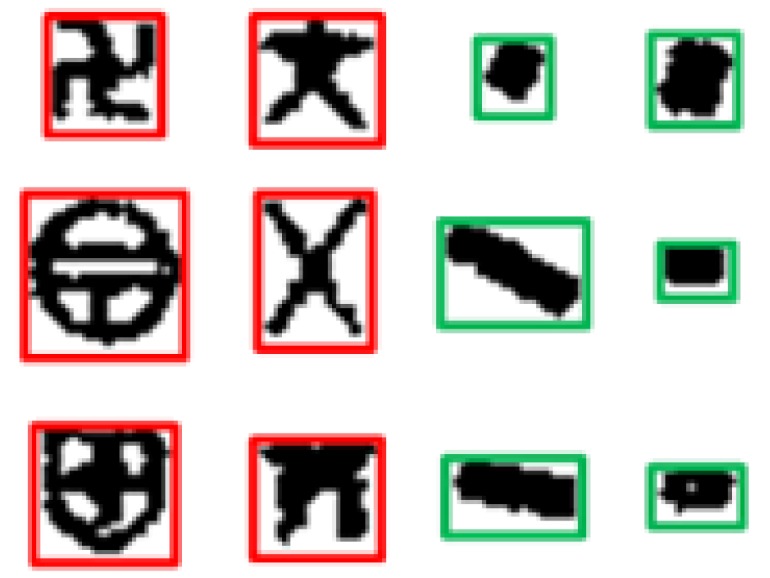
The connected region of suspected point symbols.

Size is a criterion as the threshold to distinguish the point symbols and the non-point symbols. Based on the above method of judging the minimum bounding rectangle, the connected region of suspected point symbols shown in Figure 5 can be obtained.

### 3.2. The Point Symbol Recognition Based on AlexNet

**Figure 6:**
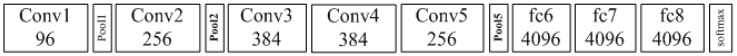
The structure of the AlexNet model.

To best of our knowledge, this is the first work in which deep transfer learning is applied to point symbol recognition and obtains a good result. The current works only process a single point symbol once, so it is inefficient to handle a large number of different point symbols in the topographic map. However, learning and training with deep transfer learning, which is similar to the learning of human brain, can effectively classify a variety of point symbols after continuous learning. The AlexNet is used to train and learn the characteristic of point symbols. The AlexNet model is displayed in Figure 6, including five convolution layers, three pooling layers, and two fully connected layers. The reasons for using the AlexNet to identify point symbols are as follows:

(1) Rectified linear unitReLU activation function is introduced into AlexNet, which can add some nonlinear factors to the neural network so that the neural network can better solve the complicated problems. Compared to the tanh activation function used in LeNet network, the nonlinear ReLU function used in AlexNet replaces the linear function to simplify the calculation and reduces the training epochs. ReLU converges faster than tanh so that the efficiency improves to a certain extent, which is proved in [21].

(2) Dropout layer is introduced into AlexNet to prevent overfitting. The Dropout layer can randomly remove a portion of neurons with certain criteria that effectively control the amount of training data to prevent overfitting. Additionally, the introduction of Dropout boosts recognition accuracy of AlexNet and learning faster.

(3) It is surprising that the ability of the characteristics learning with AlexNet can handle multiple point symbols simultaneously and reduce the interference caused by human factors to the classification result. The characteristics of point symbols are automatically learned by directly importing the images of point symbols into the AlexNet model. In addition, then the characteristics of various point symbols are simultaneously obtained achieving the recognition of multiple point symbols. Moreover, the specific network structure of AlexNet reduces the computation amount of the algorithm and tolerates a certain degree of distortion of the image.

To achieve good classification results for the target dataset after training, the training set and the test set should have the same feature space and data distribution with the traditional deep learning method. In some cases, the collection of datasets is not accessible, such as point symbol recognition in topographic maps. The concept of transfer learning solves the problems of the local optimal solution and overfitting caused by missing training sample.

The deep neural network learns the target feature from a local detail to a high-level representation. Although the two similar datasets are different on a macro scale, the local features are the same, including the boundary characteristics of the image and the color spots. That is to say, the parameters of the first several layers of the classification network obtained from the two datasets are highly similar. Furthermore, the concept of deep transfer learning is proposed. In this paper, the deep transfer learning is added to the recognition task of the point symbol in the topographic map. Firstly, according to the above statement, the pretraining model of the target classification network on a similar dataset is obtained. Because it shares some local features with the target dataset, the first several layers of network parameters of the training results are close to the final value. Then, based on the similarity degree of the two datasets, the last several layers of the pretraining model are reinitialized, and the parameter fine-tuning operation is performed on the target dataset to obtain the final value. Benefitting from the learning process from similar samples, the pretraining results have been able to identify the sample details well, and the fine-tuning based on this can improve the uncertainty of the parameter optimization direction caused by different batches of training samples. On the one hand, it can avoid overfitting problems caused by too few training samples.

**Figure 7:**
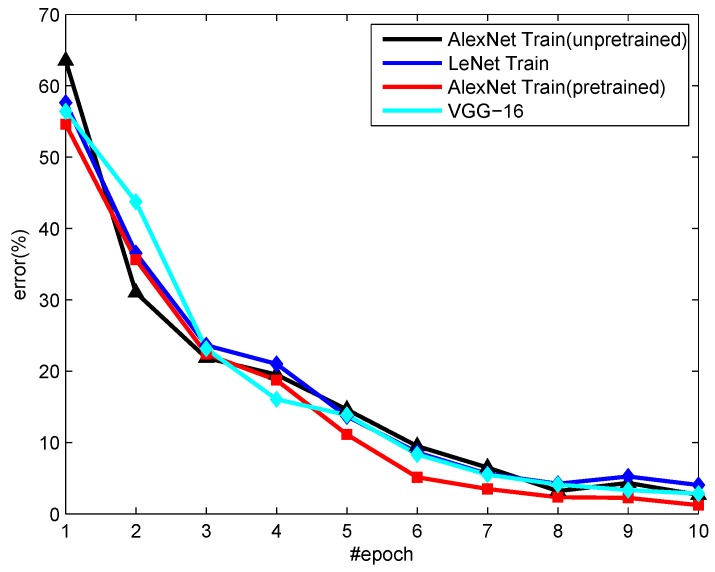
The error analysis of the different model on Point Symbols.

It can be seen from Figure 7 that the AlexNet outperforms the VGG-16 model about 1.68%. Compared with the LeNet model, it results in a small boost of 3%. In this experiment, the batch size is 50, the test_iter is 100, the learning rate is 0.001, and the training is finished after completing 10 epochs. Moreover, the pretrained AlexNet can recognize the point symbols more efficiently than the untrained AlexNet. Compared with the untrained network, the pretrained AlexNet pays more attention to the details of the point symbol and learns more deeply. Due to the particularity of the information in the topographic map, the biggest limitation of the recognition of the point symbols is that quite a few pieces of data can be obtained from the color topographic map. To solve this problem, pretraining the network based on the MNIST dataset is introduced.

Experimental results demonstrate that the effect is improved by about 3% and the recognition rate is over 98.97% when the point symbol dataset is further learned and identified based on the model that has been pretrained.

## 4. Experiment Analysis and Results

In this section, we demonstrate how our strategies work by groups of experiments. Firstly, we discuss the dataset in our experiments in Section 4.1, and then verify the effectiveness of the proposed method. Some experiments are made on several scanned topographic maps, and the proposed algorithm is compared with previous representative methods (SLS-GH method [25], BP method [14]) LeNet and VGG-16 [26] method, in Section 4.2. The runtime analysis is illustrated in the last subsection. The experiments of neural network model training and feature extracting are processed under a caffe framework on Linux Ubuntu 14.04 LTS. Others including feature extraction are implemented by Matlab R2013a (MathWorks, Natick, MA, USA) on 64-bit Windows 7 (Microsoft, Redmond, Washington DC, USA).

### 4.1. The Point Symbols Dataset

**Figure 8:**
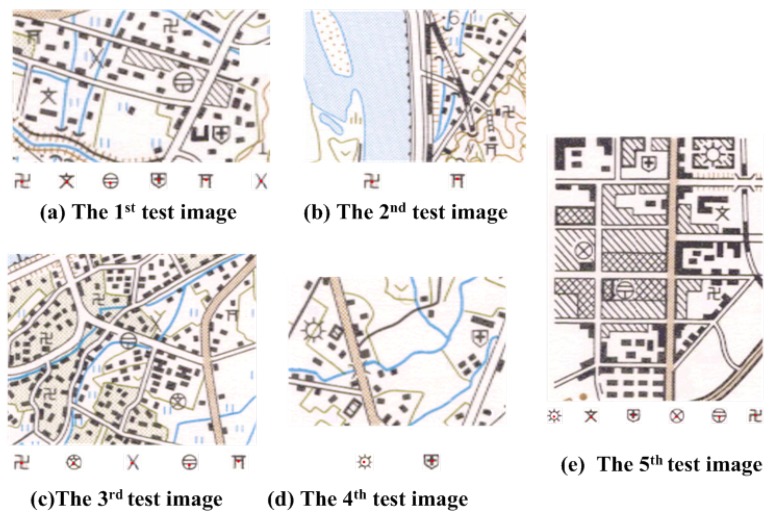
The test images for the models.

In this paper, the AlexNet trained by the MNIST dataset is used to identify the various point symbols in the color topographic map. In order to verify the validity of the proposed algorithm, five sets of test datasets are used in the experiment in Figure 8. Each of them consists of the original color topographic map, the unidentified point symbol and the referential point symbol marked in red. There are 200 sample images which are 64 × 64 in size, and nine kinds of point symbols are selected in the experiment shown in Table 1. The first 150 samples for each set are selected to be the training and the remaining images as the test data.

In this section, nine kinds of point symbols are detected directly on a color topographic map (shown in Figure 8) to verify the effectiveness of our method. As shown in Table 1, nine kinds of point symbols are defined by the map carver. The characteristic of circumscribed rectangles is regarded as the eigenvector of the point symbol approximately, and the chosen characteristics all have the invariability of universality, translation and rotation transformation.

The characteristic of the connected region of the point symbol is analyzed to obtain the circumscribed rectangle of the suspected point symbol. It can be seen from Table 2 that the length-to-width ratio of the circumscribed rectangle of the point symbol is close to 1, but the non-point symbols have the apparent difference with the length-to-width ratio of the square.

There are some challenges existing for the recognition of the point symbols such as lots of lines in color topographic maps, the complicated conditions around the symbols, and the shape features in some areas being similar to that of the point symbols. The point symbols could not be extracted completely and accurately, so prescreening before identifying the map is necessary here.

### 4.2. The Comparison of Algorithms

#### 4.2.1. The Prescreening of Point Symbols

**Figure 9:**
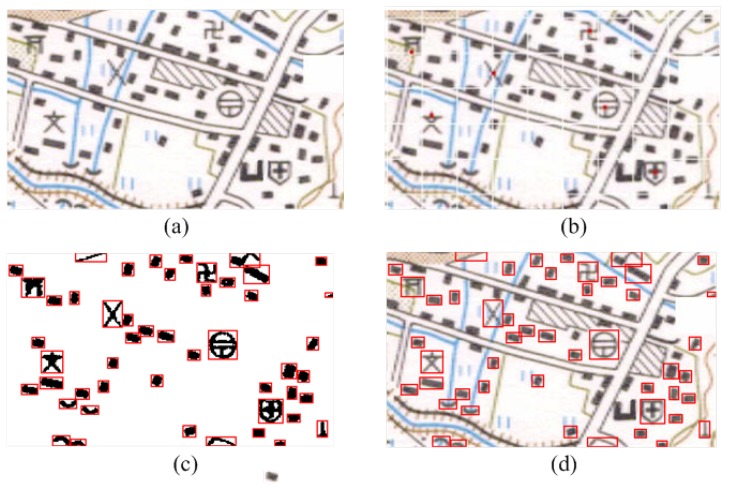
The prescreening of point symbols is presented in the figure. They are listed as (**a**) the original map; (**b**) the grid pattern; (**c**) the connected region pattern with prescreening; (**d**) the connected region pattern.

As shown in Figure 9a, there are many point symbols, which are different in shape and color information. The experimental results and data are illustrated as follows. There are many lines and background features in the topographic maps bringing challenges for recognizing the point symbols. In this paper, all of the point symbols are recognized after the prescreening based on the characteristics of point symbols.

In order to demonstrate the advantages of the proposed method further, we directly perform the map as a grid pattern directly as a comparative experiment. It can be seen that this grid pattern breaks the original shape of the point symbols, which brings challenges for symbol recognition. Point symbols are split in a grid pattern, undermining the integrity. In contrast, the extraction method based on the connected region pattern we proposed has an excellent performance.

#### 4.2.2. The Recognition of Point Symbols

In this section, our approach is performed on five scanned topographic maps, and compared with two previous representative methods, which are the traditional SLS-GH [25], BP neural networks [14], LeNet, and VGG-16 [26] methods. As shown in Table 2, there are nine-point symbols, which are different in the shape information. We use the code from the authors. The BP neural network proposed by the author refers to the concept of a multilayer perceptron. In the experiment, a 3-layer perceptron was used, with a total of 16 hidden nodes. For the BP neural network, there is no public model available to evaluate. We accomplish the code by ourselves. The experimental results and data are illustrated as follows.

**Figure 10:**
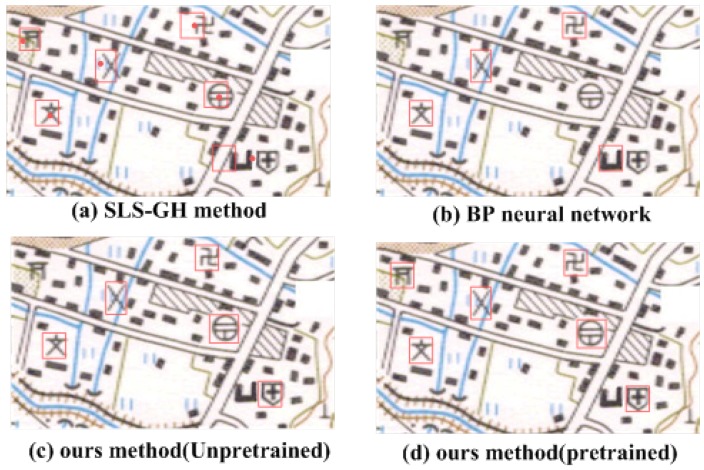
The comparison of a three recognition method.

According to the data shown, the BP neural networks method cannot detect the point symbols well. Many point symbols are recognized incorrectly, as shown in Figure 10b. In addition, there is the position error for several symbols with the traditional SLS-GH, as shown in Figure 10a. Even worse, each symbol has to be identified one by one. In addition, there are some errors with the AlexNet that is not pre-trained, which is owing to the lack of data, as shown in Figure 10c. After the AlexNet pretrained by MNIST dataset, there is a good performance, as shown in Figure 10d. There are six-point symbols in the maps, and all six point symbols have been identified.

In order to demonstrate the advantages of the proposed method further, the experiments are performed on the color topographic maps with BP neural networks method, the traditional SLS-GH, LeNet method, and the AlexNet that is not pre-trained. In order to learn the characteristics of the point symbols dataset in more depth, the initial learning rate of AlexNet and LeNet is set to 0.001 and is divided by 10 at 25% of the total number of training epochs. On the point symbols dataset, we train models for 10 epochs with a batch size of 100. The experimental data and results are illustrated in Table 3.

The result of the proposed is reported and compared with state-of-the-art methods in Table 3. Among all of the algorithms, the pretrained AlexNet network algorithm and SLS-GH algorithm obtained relatively good experimental results. The experiment shows that our method has higher recognition accuracy and less position error than other methods on the color topographic maps. Based on these experiment data, it can be seen that our method can recognize the point map symbols with higher precision. The experiment results show that the proposed method has better performance than the comparing methods, the main reason being that the proposed method introduces the deep learning based on AlexNet. Its process of characteristics learning is driven by data, which learns the target characteristics from the data directly without factitious design. Then, this way can effectively learn some characteristics ignored by humans.

### 4.3. Runtime Analysis

We compared the runtime of the proposed algorithm with BP neural network [14] and the SLS-GH method [25] and LeNet method. Bulleted lists look like this:

We report runtime of the three methods mentioned above to extract features for the entire point symbols dataset on the same PC. As shown in Table 4, the runtime of AlexNet (pretrained) is much more than the methods based on SLS-GH. It is about 4–6 times more than SLS-GH and 1–1.5 times more than the method of BP neural networks. The comparison of runtime means that our method achieves a significant speed up of runtime. It is observed that is not a fair comparison between the proposed algorithm and the SLS-GH method. The method of SLS-GH can only express the characteristics of a set of point symbols every time, which is more resource consuming. However, the proposed algorithm in this paper can learn the characteristics of various point symbols in a short period. It can be seen from Table 4 that our model considerably outperforms prior work in being able to recognize many more categories of point symbols with a large number of samples. Moreover, the model overcomes the barrier that can only identify a single point symbol and achieves the recognition of multiple point symbols simultaneously.

## 5. Conclusions

Aiming to identify the point symbols in color topographic maps, a new kind of point symbols recognition algorithm is proposed, which first introduces the deep transfer learning architecture based on AlexNet. On one hand, transfer learning is introduced in the network model training process to prevent the results from falling into local optimal solutions and speeding up the training of model parameters. Next, AlexNet is trained again based on the point symbols to learn the characteristics of point symbols further so that multiple point symbols can be handled with a high accuracy simultaneously. On the other hand, the algorithm can recognize numerous point symbols at one time while maintaining a high recognition rate, which improves the overall time efficiency indirectly.

This algorithm goes well for most point-shaped symbols that can be separated by color segmentation. However, symbols missing for most point-shaped symbols cannot be separated by color segmentation, which is the next major focus for this research.

## Figures and Tables

**Table 1:** The number of punctuation symbols in each category.

Category	1	2	3	4	5	6	7	8	9
Symbols	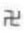	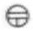	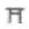	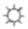	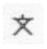	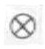	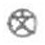	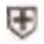	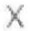
Number	250	205	200	238	240	243	204	219	212

**Table 2:** The characteristics of circumscribed rectangles of point symbols.

Category	1	2	3	4	5	6	7	8	9
Ration	1	1	0.935	0.963	0.902	0.932	0.984	0.896	0.82

**Table 3:** The comparison of the point symbol recognition.

	LeNet (Pre-Trained)	VGG-16 (Pre-Trained)	AlexNet (Not Pre-Trained)	AlexNet (Pre-Trained)	BP Networks	SLS-GH
The 1st test image	93.5	90.61	96.76	98.85	89.65	98.57
The 2nd test image	90.33	95.32	96.76	98.97	83.68	98.28
The 3rd test image	92.87	94.35	95.68	99.56	89.45	98.56
The 4th test image	92.56	96.61	94.84	98.49	81.23	97.65
The 5th test image	91.32	93.89	95.15	98.96	83.12	97.76
**Average**	92.12	94.45	95.84	**98.97**	85.43	98.16

**Table 4:** The runtime of different methods.

	AlexNet—Not Pre-Trained (ms)	BP Networks (ms)	SLS-GH (ms)
The 1st test image	256	564	890
The 2nd> test image	196	456	501
The 3rd test image	396	1064	1460
The 4th test image	231	256	328
The 5th test image	485	536	689
